# Periodontitis Severity and Subgingival Microbiome Variation in Postmenopausal Women: A Stratified Case–Control Study

**DOI:** 10.3390/life16040637

**Published:** 2026-04-10

**Authors:** Irina-Georgeta Sufaru, Stefan-Lucian Burlea, Maria-Alexandra Martu, Sorina Mihaela Solomon, Maria-Georgeta Laza, Liliana Pasarin, Elena-Odette Luca, Ioana Martu

**Affiliations:** Grigore T. Popa University of Medicine and Pharmacy, 700115 Iasi, Romania; ursarescu.irina@umfiasi.ro (I.-G.S.); sorina.solomon@umfiasi.ro (S.M.S.); laza.gina11@gmail.com (M.-G.L.); liliana.pasarin@umfiasi.ro (L.P.); elena-odette.luca@umfiasi.ro (E.-O.L.); ioana.martu@umfiasi.ro (I.M.)

**Keywords:** 16S rRNA gene sequencing, osteoporosis, periodontitis, postmenopausal women, subgingival microbiome

## Abstract

Background: This study aimed to determine whether osteoporosis is associated with differences in the subgingival microbiome of postmenopausal women, stratified by periodontitis stage. Methods: In this observational, stratified case–control study, 166 postmenopausal women were assigned to six strata defined by bone status (osteoporosis vs. normal BMD) and periodontal category (no periodontitis, Stage I–II, Stage III–IV). Standardized pooled subgingival samples were profiled by 16S rRNA gene sequencing. Community structure was evaluated using Bray–Curtis dissimilarity and tested with PERMANOVA (9999 permutations) and prespecified contrasts comparing osteoporosis versus normal BMD within each periodontal category (Holm adjustment). Alpha diversity (Shannon) was assessed using two-way ANOVA. Results: Periodontal category was strongly associated with community structure (PERMANOVA R^2^ = 0.514, pseudo-F = 86.681, *p* < 0.0001), whereas bone status (R^2^ = 0.004, *p* = 0.178) and the bone status × periodontal category interaction (R^2^ = 0.007, *p* = 0.294) were not. None of the three prespecified within-category contrasts reached significance after Holm adjustment. Shannon diversity differed by periodontal category (*p* = 1.93 × 10^−24^) but not by bone status (*p* = 0.200), with similar distributions between osteoporosis and normal BMD within each periodontal category. Conclusions: In postmenopausal women, periodontitis severity dominates variation in the subgingival microbiome, and osteoporosis does not confer an additional community-level or taxonomic signature when periodontal status is held constant. Longitudinal and multi-omic studies incorporating host-response biomarkers and therapy exposures are warranted to clarify whether osteoporosis influences periodontal susceptibility and progression primarily through host-mediated mechanisms.

## 1. Introduction

Osteoporosis is a common skeletal disorder in postmenopausal women, marked by decreased bone mineral density (BMD) and deterioration of bone microarchitecture, which increases fragility and the risk of fractures [1]. Similarly, periodontitis is a widespread chronic inflammatory disease caused by dysbiotic plaque biofilms and an abnormal host response, leading to the destruction of tissues supporting the teeth and alveolar bone loss [2]. Since both conditions mainly affect postmenopausal women and involve similar pathways of bone resorption and inflammation, their possible connection has generated ongoing interest in clinical and translational research [3].

Recent research consistently reports an epidemiological link between osteoporosis, low BMD, and adverse periodontal outcomes in postmenopausal women, though the strength of these associations and patterns of susceptibility vary across studies [4,5]. Current narrative reviews highlight that osteoporosis and periodontitis share several non-specific risk factors—such as aging, nutritional status, socioeconomic status, and medication use [6]—and common biological factors, including systemic low-grade inflammation and the receptor activator of nuclear factor kappa-B ligand/osteoprotegerin (RANKL/OPG) pathway [7,8]. These factors may jointly increase bone loss at both skeletal and alveolar sites [1]. Notably, hypoestrogenism related to menopause is increasingly recognized as a significant contributor to these connections, given estrogen’s critical roles in maintaining barrier integrity, immune function, and osteoimmunology within periodontal tissues [9].

Beyond host-focused mechanisms, the periodontal microbiome serves as a key interface connecting local inflammation with overall systemic health [10,11,12]. Periodontitis is now understood as a disease driven by microbial activity and the host response, in which changes in the composition and function of subgingival communities lead to ongoing inflammation and tissue damage [13]. Additionally, new “oral–bone” and wider oral–systemic models suggest that oral microbial imbalance can affect systemic inflammatory levels and bone-related processes, while systemic conditions can also influence the stability of the oral microbial ecosystem [14]. In this context, hormonal changes during postmenopause may create additional ecological pressures that alter microbial niches across various body sites, including the oral cavity [15].

Research on the microbiome in menopausal and bone health contexts remains inconsistent. Large studies in postmenopausal women show that endocrine factors, such as menopausal hormone therapy, can produce notable differences in subgingival microbial community structure and diversity, supporting the idea of hormone-sensitive microbial changes [16,17]. Additional evidence indicates that circulating estradiol levels may be associated with specific subgingival taxa, although much of the microbial variability is closely tied to periodontal health [18]. Moreover, metagenomic studies in postmenopausal groups have detected differences in oral microbiome composition and function across BMD levels, but these often sample multiple oral sites and may not clearly distinguish periodontal phenotype from systemic bone condition [19].

A question that remains insufficiently addressed in the available literature is whether osteoporosis is associated with a distinct subgingival microbial profile when periodontal diagnosis and severity are carefully characterized and explicitly accounted for in the study design. This distinction matters clinically, as if osteoporosis-related differences are driven primarily by periodontal stage (or ecological changes linked to menopause), then observed “bone-status effects” on microbial composition might simply be confounded by disease severity rather than indicating a direct link. To investigate this, the current study examines only postmenopausal women and uses current case definitions and staging of periodontitis [20], enabling stratified comparisons of subgingival microbial communities between osteoporosis and normal BMD groups within periodontal categories.

We designed a stratified case–control study using standardized subgingival sampling and 16S rRNA gene profiling to test the predefined hypothesis that there are no significant differences in periodontal microbial profiles between women with osteoporosis and those with normal BMD. The comparison spanned three groups: (i) participants without periodontitis; (ii) those with Stage I–II periodontitis; and (iii) those with Stage III–IV periodontitis. By employing strict eligibility criteria, detailed periodontal phenotyping, and stratification by periodontal severity, this study aims to determine whether systemic skeletal health influences microbial profiles independently of the dominant ecological effects of periodontitis.

The null hypothesis was that, among postmenopausal women, osteoporosis is not associated with detectable differences in the subgingival periodontal microbial profile compared with normal bone mineral density, when periodontal status is held constant. Accordingly, within each periodontal category (no periodontitis, Stage I–II periodontitis, and Stage III–IV periodontitis), the osteoporosis and normal BMD groups do not differ significantly in overall community structure (Bray–Curtis beta diversity) or taxonomic composition.

## 2. Materials and Methods

### 2.1. Study Design

This observational study used a stratified group-control design to evaluate the subgingival microbiome of postmenopausal women by (i) bone status (osteoporosis vs. normal bone mineral density) and (ii) periodontal category (no periodontitis vs. Stage I–II vs. Stage III–IV). The study procedures included (1) clinical screening and consent, (2) dual-energy X-ray absorptiometry (DXA), (3) full-mouth periodontal examination, and (4) standardized subgingival biofilm sampling for 16S rRNA gene sequencing.

The study protocol was approved by the Institutional Review Ethics Committee of Grigore T. Popa University of Medicine and Pharmacy in Iasi, Romania (approval no. 406, dated 6 March 2024). All participants provided written informed consent before enrollment. Data were anonymized, securely stored on password-protected systems, and managed in compliance with applicable data protection laws.

Participants were not matched on an individual basis; rather, stratification by periodontal category enabled balanced comparisons between bone-status groups within each disease stratum.

### 2.2. Participants

**Figure 1:**
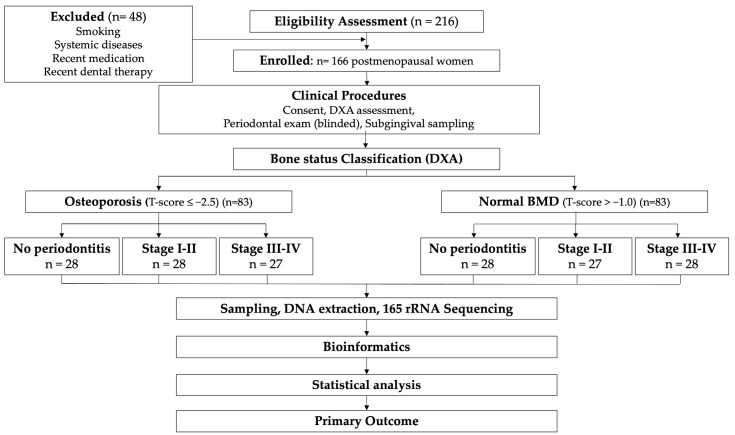
Study flowchart.

Participants were recruited between April 2024 and February 2025, from the Department of Periodontology, Grigore T. Popa University of Medicine and Pharmacy, Iași, Romania. Postmenopausal women (≥12 months of amenorrhea), with ≥20 natural teeth, presenting for routine dental or periodontal evaluation were screened for eligibility by a trained research coordinator. A total of 214 women were initially assessed: 29 were excluded based on medical history and medication criteria (systemic diseases, n = 12; medication exposure, n = 10; smoking history, n = 7), and 19 declined participation or did not complete all study procedures. The remaining 166 participants fulfilled all inclusion criteria, provided complete clinical, DXA, and microbiome data, and constituted the final analytical sample, with no post-enrollment exclusions (Figure 1).

To minimize confounding, participants were excluded if any of the following applied:

• Smoking history: any current or former smoking.
• Systemic disease: any relevant systemic condition that may affect periodontal inflammation, immunity, bone metabolism, or the oral microbiome, including diabetes/prediabetes, autoimmune/inflammatory disorders, chronic kidney disease, chronic liver disease, malignancy, inflammatory bowel disease, or thyroid/parathyroid disorders affecting bone metabolism.
• Medication exposure: systemic antibiotics within the previous 3 months; systemic corticosteroids, immunosuppressants, chemotherapy, or biologic agents within the previous 6 months; antiresorptive or anabolic osteoporosis therapies (e.g., bisphosphonates, denosumab, teriparatide, SERMs) or systemic hormone replacement therapy within the previous 12 months.
• Oral/dental interventions: periodontal therapy (subgingival instrumentation or periodontal surgery) within the past 6 months; professional dental prophylaxis within the past 4 weeks.
• Antiseptic agents: use of antiseptic mouth rinses (e.g., chlorhexidine, essential oils) within the previous 4 weeks.
• Orthodontic appliances, or oral infections other than periodontitis.

Eligibility with respect to each exclusion criterion was verified during the screening visit using one of two complementary approaches. Systemic factors—including smoking history, menopausal status, systemic disease history, medication exposure within the specified washout windows, recent periodontal treatment, dental prophylaxis, and antiseptic mouthrinse use—were ascertained through a structured, interviewer-administered medical and dental history questionnaire completed by the research coordinator. Participants were encouraged to present supporting documentation (e.g., prescription records, referral letters, specialist reports) to corroborate self-reported conditions and medication timelines.

Oral and dental factors—namely, the presence of orthodontic appliances and active oral infections other than periodontitis—were verified by direct intraoral examination performed by the calibrated periodontist. A supplementary section of the same questionnaire recorded sociodemographic information and oral hygiene behaviors. Height and weight were measured at the screening visit to calculate body mass index (BMI).

### 2.3. Bone Mineral Density Assessment and Bone-Status Classification

Bone mineral density was measured by DXA using a GE Lunar iDXA system (GE HealthCare, Madison, WI, USA), following the manufacturer’s calibration procedures. Osteoporosis was defined as a T-score ≤ −2.5 at the lumbar spine, total hip, or femoral neck. Normal bone mineral density (normal BMD) was defined as a T-score > −1.0 at all measured sites. DXA was performed within 3 months of the periodontal examination.

### 2.4. Periodontal Examination and Diagnostic

A full-mouth periodontal examination was performed by a single calibrated periodontist, blinded to bone mineral density status, using a UNC-15 manual periodontal probe (Hu-Friedy Mfg. Co., LLC, Chicago, IL, USA). Measurements were recorded at six sites per tooth (mesiobuccal, midbuccal, distobuccal, mesiolingual, midlingual, distolingual). Intra-examiner reliability was assessed in 10% of participants who were re-examined within 7–10 days. Calibration was considered acceptable if the intraclass correlation coefficient (ICC) for PPD and CAL was ≥0.80 and if ≥90% of repeated measurements were within ±1 mm.

The following clinical parameters were recorded:

• probing pocket depth (PPD; mm),
• clinical attachment level (CAL; mm),
• bleeding on probing (BOP; percentage of sites) [21],
• plaque accumulation assessed using the O’Leary Plaque Control Index [22].

Periodontitis was diagnosed and staged according to the 2017 World Workshop framework [20]. Based on this classification, participants were assigned to one of three periodontal categories:

• No periodontitis.
• Stage I–II periodontitis (mild/moderate).
• Stage III–IV periodontitis (severe).

Participants were classified into six prespecified groups based on bone status and periodontal category:

• Osteoporosis + No periodontitis.
• Osteoporosis + Stage I–II periodontitis.
• Osteoporosis + Stage III–IV periodontitis.
• Normal BMD + No periodontitis.
• Normal BMD + Stage I–II periodontitis.
• Normal BMD + Stage III–IV periodontitis.

### 2.5. Subgingival Plaque Sampling

Subgingival plaque sampling was performed by the same calibrated periodontist who carried out the periodontal examination, immediately after the clinical assessment and before any prophylaxis. Participants refrained from toothbrushing, eating, and drinking (except water) for ≥2 h before sampling.

Four sites per participant were sampled using a standardized protocol of the deepest pocket in each quadrant (one site per quadrant). If a quadrant had no site with PPD ≥4 mm (typically in participants without periodontitis), the mesiobuccal sulcus of the first molar (or the most posterior available tooth) was sampled to maintain a fixed number of sites.

Supragingival plaque was gently removed with a sterile curette without entering the sulcus. The tooth was isolated with cotton rolls and gently air-dried. Sterile endodontic paper points (ISO size 30; DiaDent Group International Inc., Burnaby, BC, Canada) were inserted into the pocket to the depth for 20 s [23]. Paper points from the four sites were pooled per participant into a sterile 1.5 mL microcentrifuge tube (Eppendorf SE, Hamburg, Germany). Samples were transported on ice to the laboratory within 2 h and stored at −80 °C until DNA extraction.

To monitor contamination, the following controls were run alongside clinical samples:

• field blanks (unused paper points handled chairside),
• extraction blanks (no-template controls) for each extraction batch.

Because a single operator collected all samples following a standardized protocol—including fixed paper-point specifications, uniform insertion time, a constant number of sampled sites per participant, and a pre-defined site-selection algorithm—inter-operator variability does not apply. Intra-operator sampling consistency was ensured through adherence to this protocol rather than through repeated same-site sampling, which would disturb the subgingival biofilm and compromise sample independence.

### 2.6. DNA Extraction and DNA Quantification

DNA was extracted using the QIAamp DNA Mini Kit (QIAGEN GmbH, Hilden, Germany). Mechanical lysis was performed with a TissueLyser II (QIAGEN GmbH, Hilden, Germany) using 0.1 mm zirconia/silica beads, bead-beating at 25 Hz for 2 × 2 min, followed by purification with the kit according to the manufacturer’s instructions.

DNA quantity and purity were measured using a NanoDrop One spectrophotometer (Thermo Fisher Scientific, Waltham, MA, USA) and a Qubit 4 Fluorometer with the dsDNA HS Assay Kit (Thermo Fisher Scientific, Waltham, MA, USA). Extracted DNA was stored at −20 °C until amplification.

All samples were identified by anonymized codes, and laboratory personnel involved in DNA extraction, library preparation, and sequencing were blinded to participants’ bone status and periodontal classification.

### 2.7. 16S rRNA Gene Amplification, Library Preparation, and Sequencing

The V3–V4 region of the bacterial 16S rRNA gene was amplified with Illumina overhang adaptors using primers 341F (5′-CCTACGGGNGGCWGCAG-3′) and 806R (5′-GACTACHVGGGTATCTAATCC-3′). PCR was performed on a Veriti 96-Well Thermal Cycler (Applied Biosystems, Thermo Fisher Scientific, Waltham, MA, USA) with KAPA HiFi HotStart ReadyMix (Roche Sequencing Solutions, Wilmington, MA, USA) and 10 ng of template DNA in 25 µL reactions. Cycling conditions were: 95 °C for 3 min; 25 cycles of 95 °C for 30 s, 55 °C for 30 s, and 72 °C for 30 s; and a final extension at 72 °C for 5 min.

Amplicons were verified by agarose gel electrophoresis, purified with AMPure XP magnetic beads (Beckman Coulter, Brea, CA, USA), and indexed with the Nextera XT Index Kit v2 (Illumina, San Diego, CA, USA). Libraries were quantified with the Qubit dsDNA HS assay, normalized to equimolar concentrations, pooled, and sequenced on an Illumina MiSeq platform (Illumina, San Diego, CA, USA) with a MiSeq Reagent Kit v3 (600-cycle), producing 2 × 300 bp paired-end reads. A PhiX control library (Illumina, San Diego, CA, USA) was added at 10%.

### 2.8. Bioinformatics Processing

Demultiplexed reads were processed in QIIME 2 (version 2024.2). Primer trimming, quality filtering, denoising, and chimera removal were performed using DADA2, resulting in amplicon sequence variants (ASVs). Taxonomy was assigned with a naïve Bayes classifier trained on the SILVA reference database (release 138.1), trimmed to the V3–V4 region. Features assigned to mitochondria/chloroplast and sequences unassigned at the domain level were removed. Samples with a sequencing depth < 10,000 reads were excluded. To reduce background contamination, negative controls were included, and contaminant features were identified and removed using the decontam prevalence method (threshold 0.50). For diversity analyses, samples were rarefied to a uniform depth of 20,000 reads per sample, as indicated by the rarefaction curves.

### 2.9. Outcomes

The primary endpoint was the overall subgingival community structure, assessed using beta diversity based on Bray–Curtis dissimilarity at the ASV level. Secondary outcomes included alpha diversity metrics, such as Observed ASVs and the Shannon index, as well as differential taxon abundances across groups.

### 2.10. Power Analysis and Sample Size

The primary comparison of interest was the difference in subgingival community structure (Bray–Curtis dissimilarity) between osteoporosis and normal BMD groups within each of the three periodontal categories, tested using PERMANOVA. Because closed-form power calculations for PERMANOVA are not available, sample size estimation was guided by published simulation-based recommendations for multivariate community analyses, which suggest that 20–30 subjects per group can detect moderate effect sizes (R^2^ ≈ 0.05–0.10) with approximately 80% power at a two-sided α of 0.05 when between-group dispersion is moderate. Accordingly, a target of 25 analyzable participants per group (total n = 150 across six strata; 50 per periodontal category) was established.

A 10% recruitment buffer was applied to accommodate potential exclusions, yielding an enrollment target of approximately 166 participants. All 166 enrolled women completed the protocol and were retained in the final analysis.

We acknowledge that the study was not separately powered for secondary outcomes, including alpha diversity comparisons and individual taxon-level differential abundance analyses, and these results should be interpreted accordingly.

### 2.11. Statistical Analysis

All statistical analyses were performed in R (version 4.3.2, R Foundation for Statistical Computing, Vienna, Austria). Continuous variables were summarized as mean ± SD or median (IQR), and categorical variables as n (%). Normality was assessed using the Shapiro–Wilk test. Clinical variables were compared across the six groups using one-way ANOVA with Tukey post hoc testing or Kruskal–Wallis with Dunn post hoc testing, as appropriate, and categorical variables were compared using Fisher’s exact test.

Before microbiome analysis, three primary planned contrasts were defined to isolate the association of osteoporosis with the subgingival microbiome within each periodontal category:

(i) osteoporosis vs. normal BMD among participants without periodontitis;

(ii) osteoporosis vs. normal BMD among participants with Stage I–II periodontitis; and

(iii) osteoporosis vs. normal BMD among participants with Stage III–IV periodontitis.

The primary endpoint (Bray–Curtis community structure) was tested using PERMANOVA (adonis2, vegan package) with 9999 permutations using the model: Bray–Curtis~bone_status × periodontal_category.

The interaction between bone_status and periodontal_category indicated that the association between osteoporosis and community structure varies across periodontal categories. If this interaction was not significant, the main effect of bone_status was interpreted as the overall difference between osteoporosis and normal BMD across periodontal categories. PERMANOVA results were reported using pseudo-F statistics, explained variance (R^2^), and *p*-values from permutation tests.

Within each periodontal category, the three planned contrasts were evaluated using PERMANOVA with the Bray–Curtis model~bone_status. Family-wise error rate across these tests was controlled using the Holm–Bonferroni procedure (two-sided α = 0.05), and the adjusted *p*-values are reported.

Prespecified sensitivity PERMANOVA models will also adjust for plaque index and bleeding on probing (BOP): Bray–Curtis~bone_status × periodontal_category + plaque_index + BOP. To further assess the robustness of the findings to residual confounding, an extended sensitivity PERMANOVA model was performed, incorporating age, years since menopause, BMI, plaque index, and BOP as covariates alongside the bone status × periodontal category terms. Confounder balance between the osteoporosis and normal BMD groups within each periodontal category was evaluated using standardized mean differences (SMDs), with SMDs < 0.40 considered indicative of adequate balance.

Alpha diversity metrics (observed ASVs and the Shannon index) were analyzed with linear models that included bone_status, periodontal_category, and their interaction. Sensitivity analyses also controlled for the plaque index and BOP. Post hoc comparisons were performed with Holm correction within each test family.

Differential abundance was assessed with ANCOM-BC2 (version 2.6.0) using non-rarefied ASV counts, with a primary focus on three predefined contrasts within periodontal categories. To control for multiple testing across taxa, the Benjamini–Hochberg false discovery rate (FDR) was applied, with *q* < 0.05 considered statistically significant. Effect estimates, including log fold changes with 95% confidence intervals and *q*-values, were reported. Community structure was examined using principal coordinates analysis (PCoA).

## 3. Results

### 3.1. Participants

A total of 166 postmenopausal women were included in the final analysis. Participants were distributed across six prespecified strata defined by bone status and periodontal category: Normal BMD/no periodontitis (n = 28), osteoporosis/no periodontitis (n = 28), Normal BMD/Stage I–II (n = 27), osteoporosis/Stage I–II (n = 28), Normal BMD/Stage III–IV (n = 28), and osteoporosis/Stage III–IV (n = 27). Overall, the mean age was 61.92 ± 5.10 years, the mean BMI was 26.19 ± 2.79 kg/m^2^, and participants had 24.32 ± 2.22 teeth.

### 3.2. Clinical and Sequencing Characteristics

Demographic, DXA, periodontal, and sequencing characteristics across the six groups are shown in Table 1. As expected, periodontal clinical parameters increased stepwise with periodontitis category: mean PPD rose from ~2.33 mm in the no-periodontitis groups (2.32 ± 0.20 Normal BMD; 2.34 ± 0.23 osteoporosis) to ~3.28 mm in Stage I–II (3.30 ± 0.24 Normal BMD; 3.26 ± 0.32 osteoporosis) and ~4.67 mm in Stage III–IV (4.65 ± 0.53 Normal BMD; 4.69 ± 0.52 osteoporosis).

A similar gradient was observed for CAL (no periodontitis: 0.65 ± 0.49 and 0.56 ± 0.43 mm; Stage I–II: 2.03 ± 0.64 and 2.12 ± 0.59 mm; Stage III–IV: 4.56 ± 0.84 and 4.15 ± 0.68 mm) and for BOP (no periodontitis: 12.36 ± 7.75% and 16.67 ± 7.28%; Stage I–II: 35.56 ± 10.33% and 39.00 ± 10.64%; Stage III–IV: 58.38 ± 10.62% and 59.10 ± 9.89%).

DXA values clearly separated bone-status strata (e.g., lumbar spine T-score ~0.0 in normal BMD vs. ~−2.9 in osteoporosis; Table 1). Sequencing depth was comparable across groups, with an overall median of 43,148 reads (IQR 34,892–51,395).

### 3.3. Community-Level Microbiome Analysis (Primary Endpoint)

Global PERMANOVA on Bray–Curtis dissimilarities showed that periodontal category explained a substantial proportion of variation in community structure (R^2^ = 0.5140, pseudo-F = 86.681, *p* < 0.0001; Table 2).

**Figure 2:**
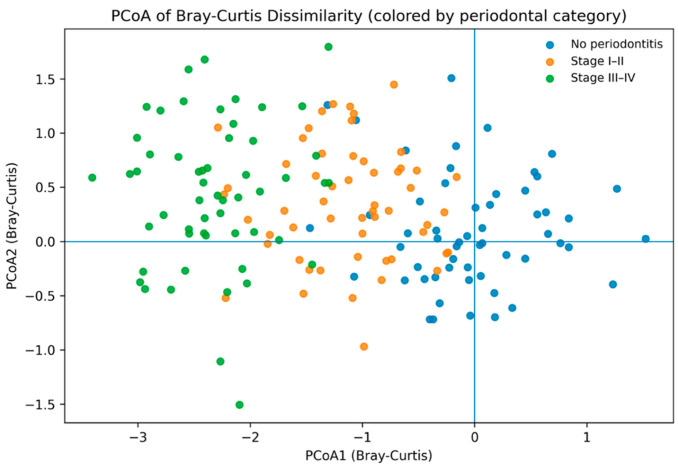
Principal Coordinates Analysis (PCoA) of Bray–Curtis dissimilarity. Each point represents one participant, colored by periodontal category. PCoA axis 1 and axis 2 represent the first and second principal coordinates, respectively. Ellipses indicate 95% confidence regions.

Bone status was not significantly associated with community structure (R^2^ = 0.0047, *p* = 0.1777), nor was the bone status × periodontal category interaction (R^2^ = 0.0070, *p* = 0.2936) (Table 2). Ordination revealed clustering primarily by periodontal category (Figure 2).

### 3.4. Primary Planned Contrasts

Among the three predefined primary contrasts comparing osteoporosis with normal BMD within each periodontal category, no significant differences in community structure were observed after Holm adjustment (Table 3). These findings support the prespecified null hypothesis of no osteoporosis-associated shift in the microbiome within periodontal strata.

### 3.5. Alpha Diversity

**Figure 3:**
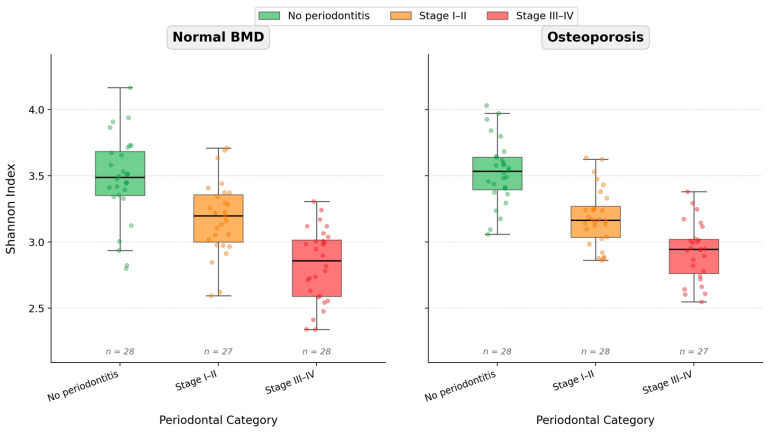
Shannon alpha diversity index stratified by bone status and periodontal category. Box plots display the median (horizontal line), interquartile range (box), and range (whiskers); individual data points are overlaid. Panels are arranged by bone status (Normal BMD, Osteoporosis), with periodontal categories (No periodontitis, Stage I–II, Stage III–IV) shown within each panel.

Alpha diversity (Shannon index) varied by periodontal category (two-way ANOVA: *p* = 1.934 × 10^−24^), with no significant main effect of bone status (*p* = 0.2003) and no bone status × periodontal category interaction (*p* = 0.636). Within each periodontal category, Shannon distributions were similar between osteoporosis and normal BMD (Figure 3).

Observed ASVs showed a comparable pattern: a significant main effect of periodontal category (two-way ANOVA: F(2,160) = 35.063, *p* = 2.36 × 10^−13^), no significant effect of bone status (F(1,160) = 0.172, *p* = 0.682), and a non-significant interaction (F(2,160) = 2.875, *p* = 0.059). Within each periodontal category, observed ASV counts were similar between osteoporosis and normal BMD groups (No periodontitis: 257.4 ± 38.5 vs. 261.3 ± 35.9; Stage I–II: 223.9 ± 30.7 vs. 243.7 ± 39.6; Stage III–IV: 198.4 ± 29.1 vs. 186.1 ± 35.7).

### 3.6. Taxonomic Composition (Secondary Analysis)

Differential abundance testing with ANCOM-BC2 (version 2.6.0), applied to non-rarefied ASV-level counts, identified ten taxa significantly associated with periodontal category after Benjamini–Hochberg FDR correction (*q* < 0.05; Table 4). Disease-associated genera—including Porphyromonas (lfc = 1.952), Tannerella (lfc = 2.067), Treponema (lfc = 1.604), and Fusobacterium (lfc = 1.237)—were enriched in Stage III–IV relative to no periodontitis, while health-associated genera such as Streptococcus (lfc = −2.004), Actinomyces (lfc = −1.608), and Neisseria (lfc = −1.586) were depleted. Veillonella was the only taxon that did not reach significance (*q* = 0.373). For the three predefined within-category contrasts (osteoporosis vs. normal BMD), no individual taxa reached significance after FDR correction in any periodontal category (all *q* > 0.05), consistent with the primary community-level findings.

### 3.7. Sensitivity Analyses

Prespecified sensitivity models, additionally adjusting for plaque index and BOP, confirmed the robustness of the primary findings. For the Shannon index, periodontal category remained significant (F(2,158) = 8.697, *p* = 2.61 × 10^−4^), while bone status (F(1,158) = 0.676, *p* = 0.419) and the interaction (F(2,158) = 0.438, *p* = 0.646) were not. Results for Observed ASVs were concordant: periodontal category (F(2,158) = 4.922, *p* = 0.008), bone status (F(1,158) = 0.003, *p* = 0.958), and interaction (F(2,158) = 2.059, *p* = 0.131). These findings indicate that the conclusions are not materially altered by adjustment for clinical inflammatory and hygiene covariates.

The sensitivity PERMANOVA model additionally adjusting for plaque index and BOP confirmed the primary findings (Table 5). Periodontal category remained the only significant predictor of community structure (pseudo-F = 13.661, *p* = 0.001), while bone status (pseudo-F = 0.317, *p* = 0.847), the interaction (pseudo-F = 1.167, *p* = 0.317), plaque index (pseudo-F = 0.598, *p* = 0.611), and BOP (pseudo-F = 0.598, *p* = 0.610) were not significant. For alpha diversity, models adjusting for plaque index and BOP yielded concordant results: the Shannon index showed a significant effect of periodontal category (F(2,158) = 8.697, *p* = 2.61 × 10^−4^) but not of bone status (*p* = 0.419) nor the interaction (*p* = 0.646), and observed ASVs followed the same pattern (periodontal category: *p* = 0.008; bone status: *p* = 0.958; interaction: *p* = 0.131). These findings indicate that adjustment for clinical covariates does not materially alter the conclusions of the primary analyses.

An extended sensitivity PERMANOVA, additionally adjusting for age, years since menopause, and BMI, further confirmed these findings (Table 6). Periodontal category remained the only significant predictor (pseudo-F = 13.456, *p* = 0.001), while bone status (pseudo-F = 0.379, *p* = 0.801), the interaction (pseudo-F = 1.421, *p* = 0.207), and all individual covariates were non-significant. Confounder balance assessment showed adequate balance (SMD < 0.40) for most variables between bone status groups within each periodontal category, with moderate imbalances observed for years since menopause in Stage I–II (SMD = −0.70) and BOP in no-periodontitis (SMD = 0.57); adjustment for these variables did not alter the conclusions.

## 4. Discussion

In this cohort of 166 postmenopausal women stratified by periodontal diagnosis and bone status, the primary signal in the subgingival microbiome aligned with periodontal disease category rather than skeletal status. At the community level, periodontal category accounted for most of the variation in Bray–Curtis dissimilarity (PERMANOVA R^2^ = 0.514, *p* < 0.0001), whereas osteoporosis status explained a negligible proportion of variance (R^2^ = 0.004, *p* = 0.178), and the bone status × periodontal category interaction was not significant (R^2^ = 0.007, *p* = 0.294).

The clear division by periodontal category corresponds with the views of periodontitis as a dysbiosis-driven inflammatory disease [24]. In this condition, local ecological changes—such as increased inflammation, deeper pockets, altered oxygen levels, and nutrient variations—change the subgingival environment, promoting the growth of pathobionts [25]. Our results also align with studies in postmenopausal women, which show that periodontal health and periodontitis are associated with significant differences in subgingival microbial communities [26]. Additionally, longitudinal data suggest that initial subgingival taxa can predict future alveolar bone loss in postmenopausal women [27]. Overall, these findings support the idea that local inflammation and niche alterations are primary factors driving microbial community changes, even in populations such as postmenopausal women where systemic bone remodeling is also important.

At the taxonomic level, genera traditionally linked to periodontal dysbiosis, such as Porphyromonas, Tannerella, Treponema, and Fusobacterium, were among the most strongly associated with the periodontal category after FDR correction. This pattern aligns with biological expectations, as these taxa are often found in more severe disease cases and can engage in polymicrobial synergy and immune evasion, thereby increasing inflammation and tissue damage [28]. Importantly, since 16S profiles usually offer genus-level resolution for many oral bacteria, interpretation should focus on overall community changes and ecological patterns rather than on individual “causative” species.

The three primary contrasts—osteoporosis versus normal BMD within (i) no periodontitis, (ii) Stage I–II periodontitis, and (iii) Stage III–IV periodontitis—showed no significant differences in community structure after Holm correction. Even in Stage III–IV, where the effect estimate was the highest (R^2^ = 0.039), the adjusted results remained non-significant. This supports the original hypothesis of no difference and indicates that, with strict exclusion criteria and standardized sampling, osteoporosis status alone may not be associated with a detectable change in subgingival community composition when the periodontal diagnosis is consistent.

This finding is consistent with previous research involving postmenopausal women, which indicated that periodontal health and microbial patterns do not necessarily vary directly with osteoporosis status [29]. It also addresses a common debate: while epidemiological studies and meta-analyses often find links between osteoporosis (or low BMD) and the severity of periodontitis or tooth loss, microbiome differences are not always consistently observed after accounting for confounding factors and disease characteristics. A plausible explanation is that osteoporosis may affect susceptibility, disease progression, or the host response (such as inflammatory amplification and regulation of bone turnover), rather than creating a distinct microbial community signature in dental plaque.

Alpha diversity (Shannon) showed significant differences across various periodontal categories (two-way ANOVA, *p* = 1.93 × 10^−24^), with no main effect of bone status (*p* = 0.200) or interaction (*p* = 0.636). The literature presents mixed findings on how alpha diversity changes with periodontitis severity, which are often influenced by sampling methods, sequencing techniques, bioinformatics approaches, and case definitions. Some studies report increased richness and diversity in inflammation-affected pockets due to niche expansion and colonization [30,31], while others observe decreased diversity, indicating the dominance of anaerobic, disease-associated bacteria [32]. Importantly, in this cohort, alpha diversity correlated more with periodontal phenotype than with skeletal phenotype. Sensitivity analyses additionally adjusting for plaque index and BOP yielded concordant results for both Shannon and Observed ASVs, supporting the robustness of these findings to potential confounding by oral hygiene and gingival inflammation.

Several explanations, not mutually exclusive, could account for the lack of detectable microbiome differences associated with osteoporosis, despite a plausible link between bone health and periodontal disease. Subgingival microbial communities are mainly shaped by local ecological factors—such as oxygen levels, the biochemical environment of gingival crevicular fluid, other medications, and, importantly, inflammation [33]. Therefore, once the periodontal condition is controlled, systemic variations in BMD may be too distant to directly influence microbial composition. It is also possible that osteoporosis and hypoestrogenism mainly affect host pathways rather than altering the microbial community itself; changes in osteoimmunologic signaling, including shifts in the RANKL/OPG axis and changes in inflammatory cytokine levels, could increase tissue vulnerability and lead to greater tissue destruction without necessarily altering which microbes are present.

Additionally, systemic BMD, as measured by DXA, may not reflect the microarchitecture and remodeling of alveolar bone or the history of periodontal breakdown at specific sites, suggesting that microbiome signals may be more closely related to local inflammation, pocket depth, and cumulative attachment loss than to skeletal T-scores. Lastly, the strict exclusion criteria—especially the removal of smokers and individuals with systemic comorbidities—aim to prevent shared risk factors between osteoporosis and periodontal disease. While this enhances internal validity, it may also reduce the observable differences between groups that might appear in more diverse, real-world populations.

A key strength of this study is its focus solely on postmenopausal women, which minimizes hormonal differences and improves the interpretability within a clinically relevant risk population. Periodontal diagnosis and severity were assessed using a staging system aligned with the 2017 World Workshop framework, ensuring current case definitions and comparability with recent studies [20]. Moreover, the analytical approach was established beforehand through predefined stratification and primary comparisons, minimizing the risk of data-driven conclusions. The technical aspects were also carefully managed, with consistent sequencing depth across groups, reducing the chance that observed patterns are due to sequencing biases or other methodological issues. Overall, the stratified design effectively separates periodontal-phenotype effects from potential osteoporosis signals, especially since periodontitis severity significantly alters subgingival microbial communities, which could otherwise skew between-group comparisons.

This study has several important limitations. Its observational (case–control) design limits causal inference and does not establish temporality. 16S rRNA profiling, which is compositional and often limited to the genus level, may overlook functionally important strain-level differences, virulence genes, and metabolic capabilities. Although standardized, subgingival sampling provides a snapshot that can vary with the selected site, pocket heterogeneity, and recent oral hygiene practices. Because paper points from four sites per participant were pooled into a single sample, spatial resolution within the oral cavity is lost; this approach precludes site-specific microbial analysis and may mask differences between actively progressing and clinically stable pockets within the same individual.

The study population was recruited from a single academic center in Iasi, Romania, and the oral microbiome is shaped by geographic, dietary, ethnic, and environmental factors that may differ across populations; therefore, generalizability to other demographic or geographic contexts should not be assumed. The age window of 50–75 years and the requirement of at least 12 months of amenorrhea exclude both early menopausal women and those older than 75, who may harbor distinct microbiome–bone interactions not captured in the present cohort. The stringent exclusion of smokers and individuals with prevalent comorbidities such as diabetes and autoimmune disorders, while designed to enhance internal validity, produces a highly selected cohort that does not reflect the clinical heterogeneity encountered in routine practice; therefore, this trade-off between internal validity and external applicability should be considered when interpreting the findings.

Additionally, residual confounding from unmeasured factors—including diet, socioeconomic status, detailed oral hygiene practices, and prior undetected medication use—cannot be entirely excluded, although extended sensitivity models incorporating age, years since menopause, BMI, plaque index, and BOP yielded results concordant with the primary analysis.

Moreover, although antiresorptive and anabolic osteoporosis therapies were excluded within the 12 months preceding enrollment, lifetime exposure to bisphosphonates—which exhibit prolonged skeletal retention—was not systematically recorded, and residual effects on bone turnover markers or the local alveolar environment cannot be entirely ruled out.

Several directions merit investigation in future studies. Longitudinal designs with repeated subgingival sampling and periodontal phenotyping would clarify whether osteoporosis influences disease progression—for instance, the rate of transition from Stage I–II to Stage III–IV or the tempo of alveolar bone loss—rather than community composition at a single time point. Prospective cohorts would also allow assessment of temporal precedence between shifts in the subgingival microbiome and changes in systemic bone metabolism.

Complementing 16S rRNA profiling with shotgun metagenomics, metatranscriptomics, and targeted metabolomics would permit functional characterization of the subgingival community. Such approaches may reveal pathway-level differences—for example, in virulence-factor expression, lipopolysaccharide biosynthesis, or short-chain fatty acid metabolism—that remain invisible at the taxonomic level alone.

Incorporating host-response biomarkers, including circulating bone-turnover markers (e.g., CTX, P1NP) and gingival crevicular fluid inflammatory mediators (e.g., IL-1β, TNF-α, RANKL/OPG ratio), would help determine whether osteoporosis modulates periodontal susceptibility through altered host–microbe signaling rather than through shifts in biofilm composition. In parallel, evaluating the effects of antiresorptive therapies and menopausal hormone therapy on both the subgingival microbiome and periodontal clinical outcomes would inform clinical decision-making.

Multi-center studies recruiting across diverse geographic, ethnic, and socioeconomic contexts are needed to assess the generalizability of the present findings. Expanding the age range and relaxing selected exclusion criteria in future cohorts would also improve external validity while retaining adequate confounder control through statistical adjustment.

Overall, the current results support a model in which the severity of periodontal disease primarily influences the structure of subgingival microbial communities in postmenopausal women. Meanwhile, osteoporosis status does not appear to confer a distinct microbial signature after accounting for periodontal diagnosis. This does not rule out meaningful osteo–periodontal interactions; instead, it indicates that these interactions are likely driven by host susceptibility and the connection between bone and immune responses, rather than by large, easily observable changes in the subgingival bacterial makeup.

Taken together, our findings are consistent with and extend prior observations indicating that, when periodontal diagnosis is rigorously defined and major confounders are minimized, systemic osteoporosis does not appear to confer an additional subgingival taxonomic signature beyond the strong ecological imprint of periodontitis severity. Future longitudinal and multi-omic studies integrating host-response biomarkers and therapy exposures are warranted to determine whether osteoporosis primarily influences periodontal susceptibility and progression through host-mediated mechanisms rather than through overt shifts in subgingival microbiome.

## 5. Conclusions

In this stratified cohort of postmenopausal women, periodontal category was the dominant determinant of subgingival microbial community structure, with clear separation across no periodontitis, Stage I–II, and Stage III–IV disease.

In contrast, osteoporosis status was not associated with detectable differences in community composition within any periodontal category, and no bone status × periodontal category interaction was observed.

## Figures and Tables

**Table 1:** Demographic, clinical, and microbiome characteristics of the study population stratified by bone status and periodontal category.

Variable	No Periodontitis		Stage I–II Periodontitis		Stage III–IV Periodontitis		Overall (n = 166)
	Normal BMD	Osteoporosis	Normal BMD	Osteoporosis	Normal BMD	Osteoporosis	
	(n = 28)	(n = 28)	(n = 27)	(n = 28)	(n = 28)	(n = 27)	
Demographic characteristics							
Age (years)	60.13 ± 4.44	59.92 ± 5.89	62.08 ± 5.80	62.76 ± 4.63	63.61 ± 4.44	63.06 ± 4.41	61.92 ± 5.10
Years since menopause	10.79 ± 5.35	9.55 ± 4.46	15.07 ± 6.05	10.76 ± 6.26	12.42 ± 5.21	14.05 ± 5.95	12.08 ± 5.82
BMI (kg/m^2^)	25.92 ± 2.75	26.35 ± 1.92	25.86 ± 3.04	26.46 ± 3.09	26.90 ± 3.23	25.65 ± 2.58	26.19 ± 2.79
Dental and bone parameters							
Teeth count	24.18 ± 2.47	24.43 ± 2.35	24.63 ± 2.36	24.36 ± 2.16	24.39 ± 1.91	23.93 ± 2.18	24.32 ± 2.22
T-score (lumbar spine)	0.02 ± 0.50	−2.81 ± 0.28	0.03 ± 0.52	−2.90 ± 0.30	−0.12 ± 0.39	−2.93 ± 0.38	−1.45 ± 1.49
T-score (total hip)	−0.06 ± 0.42	−2.62 ± 0.29	−0.09 ± 0.39	−2.69 ± 0.39	−0.05 ± 0.35	−2.59 ± 0.30	−1.35 ± 1.34
Periodontal clinical parameters							
Mean PPD (mm)	2.32 ± 0.20	2.34 ± 0.23	3.30 ± 0.24	3.26 ± 0.32	4.65 ± 0.53	4.69 ± 0.52	3.42 ± 1.03
Mean CAL (mm)	0.65 ± 0.49	0.56 ± 0.43	2.03 ± 0.64	2.12 ± 0.59	4.56 ± 0.84	4.15 ± 0.68	2.34 ± 1.67
BOP (%)	12.36 ± 7.75	16.67 ± 7.28	35.56 ± 10.33	39.00 ± 10.64	58.38 ± 10.62	59.10 ± 9.89	36.72 ± 20.48
Plaque (%)	19.64 ± 9.26	23.29 ± 10.27	34.86 ± 10.10	38.47 ± 10.44	55.05 ± 12.57	50.53 ± 12.25	36.90 ± 16.85
Sequencing and diversity metrics							
Sequencing reads †	47,102 (34,269–53,704)	41,651 (34,452–47,946)	38,705 (33,182–51,744)	43,072 (37,718–48,794)	46,249 (37,429–52,940)	41,657 (35,860–47,799)	43,148 (34,892–51,395)
Shannon index	3.48 ± 0.33	3.53 ± 0.24	3.18 ± 0.28	3.19 ± 0.22	2.83 ± 0.28	2.93 ± 0.22	3.19 ± 0.37

Values are mean ± SD unless otherwise indicated. † Median (IQR). BMD, bone mineral density; BMI, body mass index; PPD, probing pocket depth; CAL, clinical attachment level; BOP, bleeding on probing.

**Table 2:** Global PERMANOVA for Bray–Curtis dissimilarity (9999 permutations).

Term	df	R^2^	Pseudo-F	*p* (Perm)
Bone status	1	0.0047	1.572	0.1777
Periodontal category	2	0.5140	86.681	0.0001
Bone × periodontal	2	0.0070	1.179	0.2936

**Table 3:** Primary planned contrasts: Osteoporosis vs. normal BMD within each periodontal category (PERMANOVA; Holm-adjusted).

Periodontal Category	N	R^2^	Pseudo-F	*p* (Perm)	*p* (Holm)
No periodontitis	56	0.0052	0.283	0.8904	0.8904
Stage I–II	55	0.0185	0.999	0.3961	0.7922
Stage III–IV	55	0.0397	2.194	0.0693	0.2079

**Table 4:** Differential taxon abundance by periodontal category (ANCOM-BC2). Log-fold changes are relative to the No Periodontitis reference group.

Taxon	Stage I–II Periodontitis vs. No Periodontitis			Stage III–IV Periodontitis vs. No Periodontitis			*q*-Value (Overall)
	lfc	95% CI	*Q*-Value	lfc	95% CI	*q*-Value	
Streptococcus	−0.976	[−1.21, −0.74]	<0.0001	−2.004	[−2.24, −1.77]	<0.0001	<0.0001
Tannerella	+0.799	[0.53, 1.07]	<0.0001	+2.067	[1.80, 2.34]	<0.0001	<0.0001
Porphyromonas	+0.960	[0.70, 1.22]	<0.0001	+1.952	[1.69, 2.22]	<0.0001	<0.0001
Actinomyces	−0.644	[−0.88, −0.41]	<0.0001	−1.608	[−1.84, −1.37]	<0.0001	<0.0001
Treponema	+0.654	[0.38, 0.93]	<0.0001	+1.604	[1.33, 1.88]	<0.0001	<0.0001
Fusobacterium	+0.836	[0.60, 1.07]	<0.0001	+1.237	[1.00, 1.47]	<0.0001	<0.0001
Neisseria	−1.014	[−1.32, −0.71]	<0.0001	−1.586	[−1.89, −1.28]	<0.0001	<0.0001
Other	−0.297	[−0.42, −0.17]	<0.0001	−0.644	[−0.77, −0.52]	<0.0001	<0.0001
Rothia	−0.394	[−0.75, −0.04]	0.0355	−1.454	[−1.81, −1.10]	<0.0001	<0.0001
Prevotella	+0.245	[0.07, 0.41]	0.0065	+0.533	[0.36, 0.70]	<0.0001	<0.0001

ANCOM-BC2, Analysis of Compositions of Microbiomes with Bias Correction 2; lfc, log-fold change relative to the No Periodontitis reference group; 95% CI, 95% confidence interval for the log-fold change; *q*-value, Benjamini–Hochberg false-discovery-rate-adjusted *p*-value. Positive lfc indicates enrichment in the comparison group relative to No Periodontitis; negative lfc indicates depletion.

**Table 5:** Sensitivity PERMANOVA for Bray–Curtis dissimilarity, additionally adjusting for plaque index and bleeding on probing (999 permutations).

Term	df	R^2^	Pseudo-F	*p* (Perm)
Bone status	1	0.0009	0.317	0.847
Periodontal category	2	0.0814	13.661	0.001
Bone × Periodontal	2	0.0070	1.167	0.317
Plaque index	1	0.0018	0.598	0.611
BOP	1	0.0018	0.598	0.610
Residual	158	0.4708		
Total	165	1.0000		

BOP, bleeding on probing. Model: Bray–Curtis~bone status × periodontal category + plaque index + BOP. *p*-value indicates statistical significance (*p* < 0.05).

**Table 6:** Sensitivity PERMANOVA, additionally adjusting for age, years since menopause, and BMI.

Term	df	R^2^	Pseudo-F	*p* (Perm)
Bone status	1	0.0011	0.379	0.801
Periodontal category	2	0.0791	13.456	0.001
Bone × Periodontal	2	0.0084	1.421	0.207
Age	1	0.0030	1.012	0.375
Years since menopause	1	0.0065	2.211	0.087
BMI	1	0.0060	2.054	0.109
Plaque index	1	0.0015	0.517	0.671
BOP	1	0.0015	0.514	0.676

## Data Availability

The processed taxonomic abundance data and deidentified clinical metadata supporting the findings of this study are available from the corresponding author upon reasonable request. Requests will be assessed in accordance with the institutional ethics approval (no. 406, dated 6 March 2024) and applicable data protection regulations. Raw 16S rRNA gene sequencing reads (FASTQ files) are retained on institutional password-protected systems.

## Abbreviations

The following abbreviations are used in this manuscript:

16S	16S ribosomal RNA gene
ANOVA	analysis of variance
ASV	amplicon sequence variant
ASVs	amplicon sequence variants
BMD	bone mineral density
BMI	body mass index
BOP	bleeding on probing
CAL	clinical attachment level
DADA2	Divisive Amplicon Denoising Algorithm 2
DNA	deoxyribonucleic acid
DXA	dual-energy X-ray absorptiometry
FDR	false discovery rate
ICC	intraclass correlation coefficient
IL-1β	interleukin 1 beta
IQR	interquartile range
MiSeq	Illumina MiSeq sequencing platform
OPG	osteoprotegerin
PCR	polymerase chain reaction
PCoA	principal coordinates analysis
PERMANOVA	permutational multivariate analysis of variance
PPD	probing pocket depth
PhiX	PhiX control library (Illumina)
QIIME	Quantitative Insights Into Microbial Ecology
RANKL	receptor activator of nuclear factor kappa-B ligand
SD	standard deviation
SILVA	SILVA ribosomal RNA gene database
TNF-α	tumor necrosis factor alpha
